# Associations of cholesterol and vitamin D metabolites with the risk for development of high grade colorectal cancer

**DOI:** 10.2478/jomb-2019-0047

**Published:** 2020-09-02

**Authors:** Sandra Vladimirov, Aleksandra Zeljković, Tamara Gojković, Milica Miljković, Aleksandra Stefanović, Dejan Zeljković, Bratislav Trifunović, Vesna Spasojević-Kalimanovska

**Affiliations:** 1 University of Belgrade, Faculty of Pharmacy, Department of Medical Biochemistry, Belgrade; 2 Military Medical Academy, Clinic for General Surgery, Belgrade

**Keywords:** 25-hydroxyvitamin D, 7-dehydrocholesterol, 24,25-dihydroxyvitamin D, total cholesterol, colorectal cancer, 25-hidroksivitamin D, 7-dehidroholesterol, 24,25-dihidroksivitamin D, ukupan holesterol, kolorektalni karcinom

## Abstract

**Background:**

Vitamin D deficiency is repeatedly reported in colorectal cancer (CRC). Since cholesterol and vitamin D share common precursor 7-dehydrocholesterol (7-DHC), it would be important to explore the associations of key vitamin D metabolites and serum lipid parameters in patients with high and low grade CRC. The aim of this study was to analyze relationships between serum 25(OH)D3, 24,25(OH)2D3 and 7-DHC levels and serum lipids in patients with CRC, and to evaluate their potential for prediction of risk for development of high grade CRC.

**Methods:**

We recruited 82 patients CRC and 77 controls. 7-DHC, 25(OH)D3 and 24,25(OH)2D3 were quantified by LC-MS/MS methods.

**Results:**

7-DHC, 25(OH)D3 and vitamin D metabolic ratio (VDMR) were significantly lower in CRC patients than in control group (P<0.001, P<0.010, P<0.050 and P<0.050, respectively). 25(OH)D3 levels were higher in patients with grade I CRC when compared to grade II (P<0.050). All vitamin D metabolites positively correlated with total cholesterol (TC) concentration in CRC patients. 25(OH)D3 was significant predictor of increased CRC risk (P<0.010). After adjustment for TC concentration, 25(OH)D3 lost its predictive abilities. However, 25(OH)D3 remained significant predictor of poorly differentiated type of cancer (P<0.050).

**Conclusions:**

We found significant positive association between vitamin D status and serum total cholesterol. Although low 25(OH)D3 was found to be a significant risk factor for CRC development, the obtained results primarily suggest profound impact of cholesterol level on vitamin D status in CRC. However, our results suggest that low 25(OH)D3 might independently contribute to development of poorly differentiated tumor.

## Abstract (sr)

**Uvod:**

Deficijencija vitamina D je učestalo javlja kod obolelih od kolorektalnog karcinoma (CRC). Kako holesterol i vitamin D dele zajednički prekursor 7-dehidroholesterol (7-DHC), bilo bi značajno ispitati povezanost ključnih metabolita vitamin D i serumskih lipidnih parametara kod pacijenata sa CRC klasifikovanih na osnovu gradusa. U ovom radu ispitivali smo odnos između serumskih koncentracija 25(OH)D3, 24,25(OH)2D3 and 7-DHC i serumskih lipida kod pacijenata sa CRC, kao i doprinos metabolita vitamina D u predikciji rizika za nastanak CRC visokog gradusa.

**Metode:**

U studiji su učestvovala 82 pacijenta sa CRC i 77 zdravih ispitanika. 7-DHC, 25(OH)D3 i 24,25(OH)2D3 su kvantifikovani pomoću metode LC-MS/MS.

**Rezultati:**

7-DHC, 25(OH)D3 i odnos metabolita vitamina D (VDMR) su bili značajno niži kod pacijenata sa CRC u odnosu na kontrolnu grupu (P<0,001, P<0,010, P<0,050 i P<0,050, redom). Koncentracije 25(OH)D3 su bile više kod pacijenata sa gradusom I u odnosu na pacijente sa gradusom II (P<0,050). Svi metaboliti vitamina D su pozitivno korelirali sa ukupnim holesterolom kod pacijenata sa CRC. 25(OH)D3 je bio značajan prediktor povećanog rizika za nastanak CRC (P<0,010). Nakon korekcije za koncentraciju ukupnog holesterola, 25(OH)D3 je izgubio prediktivni značaj. Međutim, 25(OH)D3 je ostao značajan prediktor slabo diferenciranog karcinoma (P<0,050).

**Zaključak:**

Utvrđena je značajna pozitivna korelacija iz među statusa vitamina D i serumskih koncentracija ukupnog holesterola. Iako je uočeno da je nizak 25(OH)D3 značajan faktor rizika za razvoj CRC, naši rezultati upućuju na dominantan uticaj holesterola na status vitamina D kod ovih pacijenata. Ipak, uočeno je da niski nivoi 25(OH)D3 mogu nezavisno doprineti razvoju slabo diferentovanih oblika CRC.

## Introduction

Broadly recognized prevalence of vitamin D deficiency in patients with colorectal cancer (CRC) directed scientific research towards the investigation of its role during the onset and progression of this malignancy. Anticancerous effects of vitamin D are well known and include induction of antiinflammatory, anti-oxidative, anti-proliferative, anti-apoptotic and pro-differentative properties in both normal and malignant cells [1] [2]. Yet, in spite of numerous epidemiological, clinical and interventional studies, no definitive conclusions are drawn regarding the usefulness of vitamin D treatment in CRC [3]. Furthermore, a recent Mendelian randomization study has demonstrated a lack of causality between genetically determined lower levels of vitamin D and CRC risk [4], suggesting that further research should rather focus on mechanisms that can alter vitamin D metabolism and consequently, its effects in CRC.

Many confounding factors, including sunlight exposure, obesity, body-mass index and lipid status can affect vitamin D level [5] [6]. The relationship of vitamin D with serum lipids can be particularly important, considering that 7-dehydrocholesterol (7-DHC) is a direct precursor of cholesterol, but also a direct precursor of vitamin D synthesis in the skin. It has been hypothesized that this metabolite and the activity of enzyme 7-DHC-reductase (DHCR7) might be critical points in determining both cholesterol and vitamin D levels [7], but this issue is insufficiently explored in CRC.

Metabolism of vitamin D is complex and includes several intermediate compounds that are proposed as markers of its status in the organism. Although without biological activity, 25-hydroxyvitamin D (25(OH)D3) is generally accepted as a reliable marker of vitamin D status [8]. However, recent evidences have emphasized 24,25-dihydroxyvitamin D (24,25(OH)2D3), a catabolic product with negligible biological activity, as a promising marker for prediction of vitamin D deficiency and several associated pathological conditions [9]. Accordingly, the ratio between 25(OH)D3 and 24,25(OH)2D3, or vitamin D metabolite ratio (VDMR) might provide more accurate insight into vitamin D status and more precise prediction of possible effects of disturbed vitamin D levels [10]. However, there is scarce data regarding the usefulness of VDMR in CRC patients.

The goal of this study was to estimate serum 25(OH)D3 levels and concentrations of 7-DHC and 24,25(OH)_2_D3 in patients with CRC. We analyzed mutual relationship between these metabolites and evaluated their predictive abilities, as well as possible usefulness in prediction of CRC risk. Additionally, we were interested in evaluation of the associations between vitamin D metabolites and markers of serum lipid status.

## Materials and Methods

### Subjects

For this study, we recruited 82 patients with first time diagnosed CRC. Study participants were selected from a larger cohort of 126 patients from the Clinic for General Surgery, Military Medical Academy in Belgrade, who were involved in larger research project investigating lipid status, redox balance and inflammatory markers in CRC [11] [12]. Inclusion criteria for participating in the study were: pathohistological confirmation of CRC, absence of other malignant diseases or non-malignant systemic diseases of bowel, kidney and liver, no use of hypolipidemic therapy, no use of vitamin D supplements and no use of neoadjuvant chemotherapy. After exclusion of subjects with incomplete clinical or laboratory data, the final patient's group consisted of 82 participants. Blood samples were taken immediately before surgical procedure. After an overnight fasting, blood samples were collected to EDTA plasma and serum evacuating tubes. Following the separation of serum and plasma, aliquots were frozen and stored at -80°C for further analysis.

Tumor grades were defined according to pathohistological findings as: grade I (well differentiated tumor), grade II (moderately differentiated tumor) and grade III (poorly differentiated tumor). CRC grade I was diagnosed in 31.6%, grade II in 53.2% and grade III in 15.2% of patients. For control group, we selected 78 healthy volunteers during their annual medical check-up examinations. All participants in control group had negative family anamnesis for CRC and were free of any acute or chronic disease, or use of any therapy which could affect lipid status or vitamin D levels.

Study participants were interviewed by using of standardized questionnaire. Basic information on patients' anthropometric characteristics, life style habits, use of any drugs and personal and family anamnesis were collected. Body mass index (BMI) was calculated as body weight (kg)/body height (m^2^).

The study protocol was designed according to the ethical principles of the Helsinki Declaration. Local Ethics Committee carefully examined and approved the study. All participants were informed about the study aim and design and all of them signed an informed consent prior to the involvement.

### Methods

Total proteins and components of lipid profile, including total cholesterol (TC), low-density lipoprotein cholesterol (LDL-C), high-density lipoprotein cholesterol (HDL-C) and triglycerides (TG), were determined by routine laboratory methods on ILab 300+ automatic analyzer (Instrumentation Laboratory, Diamond Diagnostics, USA). VDBP was quantified using commercial ELISA assay for human samples, VDBP Quantikine (R&D Systems).

### Vitamin D metabolites and 7-DHC analyses

Analytical HPLC grade standards were used for quantification of the 25(OH)D3, (24R)-24,25(OH)_2_D3 (Sigma-Aldrich, St. Louis, USA). Deuterated internal standards cholesterol-26,26,26, 27,27,27-d6,25(OH)D3 3-d6 and 1,25(OH)_2_D3 3-d6 were obtained from Medical Isotopes (Pelham, USA).

KOH was purchased from POCH (Center Valley, PA, USA), while ethanol, methanol, n-hexane, ethyl-acetate, methyl-tert-buthyl ether (MTBE) and acetonitrile (HPLC grade) were obtained from Fisher (Pittsburgh, PA, USA).

The analysis of vitamin D status was conducted using LC-MS/MS method developed and fully validated in our laboratory. Firstly, 500 mL of serum was placed in a reaction tube and 2.5 mL of ice cold acetonitrile were added to allow for the protein denaturation to happen. This mixture was vigorously vortexed for 30s, and centrifuged at 4000xg for 30 min. The resulting supernatant was decanted into a clean reaction tube, and evaporated under nitrogen stream to the final volume of approx. 500 mL. Afterwards, the 1 mL of HPLC-grade water was added, alongside with 2 mL of MTBE: ethylacetate mixture (9: 1, v: v). The tube was vigorously vortexed, and the upper layer was transferred into clean vial and dried under nitrogen. This extraction procedure was repeated three times in total, and all the extracts were collected in the same vial and evaporated to dryness. In the final step, the extract was reconstituted in 20 μL of methanol and injected into the LC-MS/MS instrument. Chromato-graphic separation of 25(OH)D3 and 24,25(OH)_2_D3 and corresponding internal standards was achieved in under 20 min using Kinetex F5 (150×4.6 mm, 2.6 μm, 100Å) LC column with corresponding guard column and isocratic mobile phase methanol: water (85: 15, v: v). Mobile phase flow was 0.3 mL/min and column temperature 20 °C. Quantification was done using multiple-reaction-monitoring (MRM) on triple-quad mass spectrometer Agilent 6420 equipped with MMI/APCI ion source. The following m/z transitions were used for quantification of the corresponding metabolites and internal standards: 383.3 115, 105.1 for 25(OH)D3, 381.2 14 1.1, 105.2 for 24,25(OH)_2_D3, 389.37 115.2, 105.3 for 25(OH) D3-d6 and 405.37 105 for 1,25(OH)_2_D3-d6.

7-dehydrocholesterol was quantified by another LC-MS/MS method developed and validated in our laboratory. 100 μL was mixed with internal standard and 1 mL of 2% potassium hydroxide in ethanol, vortexed and incubated for 30 min at 45 °C. Afterwards, 500 μL of water was added and 2 mL of nhexane. After vortexing and centrifugation (1500xg for 5 min), organic layer was separated. After collecting and combining three organic layers into one reaction tube, the extract was dried under nitrogen. Prior the analysis, the extract was reconstituted in 100 μL of methanol. Poroshell 120 EC-C18 column (4.6×150 mm, 2.7 μm) with corresponding guard column, and isocratic mobile phase containing acetonitrile: metha-nol: water (80: 18: 2, v: v: v) were used for chromatographic separation. 7-dehydrocholesterol and internal standard were eluted in 30 min runtime. The following m/z transitions were used for quantification of the 7-DHC and cholesterol-26,26,26,27,27,27-d6: 367.3→ 131.3, 105.3 and 375.3→ 105.2, 95.2, respectively. Quantification was done using multiplereaction-monitoring (MRM) on triple quad mass spectrometer Agilent 6420 equipped with MMI/APCI ion source.

### Statistical analysis

Normality of data was tested by the Shapiro-Wilk test. Normally distributed variables are presented as means ± standard deviation, while asymmetrically distributed data as median (interquartile range). Categorical data are presented as absolute frequencies and analyzed by the Chi-square test. For comparison of continuous data with normal and asymmetrical distribution were used Student t-test and Mann-Whitney U-test, respectively. Comparison of data stratified according to tumor grades was performed by Kruskal-Wallis test. Correlation analysis was performed by using Spearman correlation coefficients. Univariate logistic regression was employed for detection of significant predictors of CRC development and tumor grade. Adjustment in multivariate logistic regression analysis was made for age, gender and BMI (Model 1) and for age, gender, BMI and TC level (Model 2). For statistical analysis we used IBM® SPSS® model 22.0. Differences were considered significant if P <0.05.

## Results

General characteristics of the examined groups are presented in Table 1. Patients were older than controls. Nevertheless, there were no significant correlations between age and 25(OH)D3, 24,25(OH)2D3, 7-DHC, VDMR, VDBP in control group (ρ=-0.051, P=0.659; ρ=0.016, P=0.891; ρ=-0.056, P=0.632; ρ=-0.099, P=0.390; ρ=-0.172, P=0.197, respectively) nor in the patient group (ρ=-0.162, P=0.152; ρ=-0.184, P=0.102; ρ=-0.135, P=0.233; ρ=-0.111, P=0.326; ρ=-0.155, P=0.230, respectively). Additionally, we divided both cohorts (patients and controls) into quartiles according to age and also found no statistically significant differences in all of the vitamin D-related parameters between these quartiles (data not shown). Male sex was more prevalent in the CRC group. BMI and total proteins concentrations were significantly lower in CRC patients. Our results demonstrated a significant decrease in TC, LDL-C and HDL-C concentrations in patients with CRC when compared to healthy individuals. Levels of TG were comparable among the groups.

**Table 1:** General characteristics and parameters of vitamin D status in study groups Values are presented as median (interquartile range) and compared by the Mann-Whitney U-test. *Values are presented as mean ± standard deviation and compared by the Student t-test. #Values are presented as absolute frequencies and compared by the Chi-square test.

Parameter	CRC patients (N=82)	Controls (N=77)	P
Age (years)*	64.51±10.89	54.09±7.73	<0.001
Gender (male/female)#	58/24	44/33	0.098
BMI (kg/m^2^)*	25.20±3.28	26.35±3.72	0.030
Total protein (g/L)*	65.87±6.91	73.40±6.95	<0.001
TC (mmol/L)	4.43 (3.68–4.97)	5.62 (4.63–6.38)	<0.001
LDL-C (mmol/L)	2.81 (2.13–3.20)	3.64 (2.88–4.33)	<0.001
HDL-C (mmol/L)	0.99 (0.79–1.18)	1.23 (0.95–1.53)	<0.001
TG (mmol/L)	1.22 (0.99–1.43)	1.31 (1.04–1.68)	0.316
7-DHC (μmol/L)	1.24 (0.91–1.94)	1.75 (1.23–2.56)	0.002
25(OH)D3 (ng/mL)	15.93 (12.04–22.18)	18.98 (15.85–23.75)	0.003
24,25(OH)_2_D3 (ng/mL)	2.98 (2.54–3.62)	3.26 (2.70–4.00)	0.135
VDMR	5.12 (4.49–6.17)	5.55 (4.84–6.66)	0.016
VDBP (μg/mL)	265.66 (210.45–332.68)	300.56 (258.12–342.88)	0.030

Next we analyzed differences in markers of vitamin D status. Concentrations of 7-DHC were lower in CRC patients, as well as levels of 25(OH)D3. In addition, the patients had lower levels of VDBP. We did not find significant differences, although we recorded lower levels of 24,25(OH)_2_D3 in CRC group. Finally, values of VDMR were decreased in patients (Table 1).

In order to check the influence of seasonal variations and exposure to UV light on markers of vitamin D status, we divided both patients and controls in two subgroups, according to the season when blood samples were collected (winter season from November to April, or summer season from May to October). The results are presented in Table 2. None of the examined markers in patients differed with respect to season when samples are taken. On the other hand, in the control group we found significantly higher concentrations of 25(OH)D3 and 24,25(OH)_2_D3 in subjects who were included in study during the season with higher exposure to UV light.

**Table 2:** Seasonal effects on parameters of vitamin D status Values are presented as median (interquartile range) and compared by the Mann-Whitney U-test

Parameter	CRC patients		P	Controls		P
	Summer season (N= 48)	Winter season (N= 34)		Summer season (N= 23)	Winter season (N= 54)	
7-DHC (mmol/L)	1.28 (0.47–2.13)	1.24 (0.91–1.93)	0.918	1.61 (1.05–2.27)	1.90 (1.39–2.79)	0.125
25(OH)D3 (ng/mL)	16.13 (12.41–22.92)	15.98 (10.61–21.66)	0.618	22.54 (16.37–31.40)	18.33 (15.04–22.28)	0.023
24,25(OH)2D3 (ng/mL)	3.13 (2.61–4.26)	2.84 (2.61–3.67)	0.408	3.55 (3.04–5.38)	3.15 (2.62–3.74)	0.005
VDMR	5.12 (4.52–6.10)	5.09 (4.41–6.36)	0.925	5.53 (4.83–6.63)	5.70 (4.90–6.76)	0.738
VDBP (μg/mL)	266.94 (220.45–340.80)	260.13 (246.49–336.02)	0.797	334.28 (232.42–389.63)	296.60 (259.86–334.28)	0.352

Analysis of vitamin D status parameters in patients with different tumor grades (Table 3) demonstrated a decrease in concentrations of 25(OH)D3 alongside with the increase of tumor grade, with statistical significance reached between grade I and grade II. In contrast, concentration of 24,25(OH)2D3 was significantly higher in subjects with tumor grade II when compared to patients with well differentiated tumors. We found no significant differences in TC concentration among patients with tumor grades I-III.

**Table 3:** TC and parameters of vitamin D status according to CRC grade Values are presented as median (interquartile range) and compared by the Kruskal-Wallis test and post hoc Mann-Whitney test. a - significantly different from Grade I *P<0.050; **P<0.01

Parameter	Grade I	Grade II	Grade III	P
TC (mmol/L)	4.23 (3.44–5.23)	4.34 (3.75–4.80)	4.48 (3.65–5.03)	0.705
7-DHC (mmol/L)	1.25 (1.06–2.14)	1.23 (0.94–1.81)	0.79 (0.79–1.81)	0.563
25(OH)D3 (ng/mL)	19.66 (15.74–25.06)	14.77 (10.31–19.09)a**	14.27 (11.96–18.90)	<0.050
24,25-(OH)_2_D3 (ng/mL)	3.11 (2.76–4.20)	3.18 (2.50–3.45) a*	2.93 (2.61–3.86)	<0.050
VDMR	5.68 (4.66–7.18)	4.78 (3.90–5.60)	4.74 (4.46–5.20)	0.143
VDBP (μg/mL)	266.94 (223.11–361.62)	260.13 (197.57–302.90)	306.51 (208.21–366.40)	0.380

In the next step, we explored correlations of vitamin D status markers with other determined parameters. Spearman correlation analysis did not revealed any significant correlations between vitamin D status markers and anthropometric measures or protein levels (data not shown). Regarding relationship with serum lipid levels, we found significant positive correlations between TC concentration and levels of 7-DHC (ρ=0.347; P<0.01), 25(OH)D3 (ρ=0.245; P<0.05) and 24,25(OH)_2_D3 (ρ=0.295; P<0.01) in CRC patients. In addition, significant positive correlation was observed between LDL-C and 7-DHC (ρ=0.293; P<0.01) and 25(OH)D3 (ρ=0.238; P<0.05). Regarding the control group, positive association of TG concentration with 7-DHC was recorded (ρ=0.331; P<0.01).

To further explore the observed associations of TC with markers of vitamin D status, we stratified CRC patients according to median values of TC concentrations. The results of comparison of vitamin D status markers between the two groups of patients with different TC levels are presented in Table 4. We observed significantly higher levels of 7-DHC, 25(OH)D3 and 24,25(OH)2D3 in a group of CRC patients with higher concentrations of TC. On the other hand, values of VDMR and VDBP did not significantly differ among groups.

**Table 4:** Changes in parameters of vitamin D status according to TC concentration in CRC patients Values are presented as median (interquartile range) and compared by the Mann-Whitney U-test.

Parameter	TC< 4.43 mmol/L	TC> 4.43 mmol/L	P
7-DHC (mmol/L)	1.08 (0.77–1.52)	1.47 (1.01–2.33)	0.014
25(OH)D3 (ng/mL)	15.67 (10.07–16.68)	17.23 (12.21–24.90)	0.020
24,25(OH)2D3 (ng/mL)	2.82 (2.44–3.44)	3.36 (2.69–3.93)	0.006
VDMR	4.94 (4.50–5.58)	4.98 (4.43–6.77)	0.636
VDBP (μg/mL)	263.11 (209.07–326.94)	269.06 (210.77–343.53)	0.741

Finally, we examined a capacity of explored vitamin D metabolites and VDBP for possible use as markers of CRC risk prediction (Table 5). Univariate logistic regression analysis revealed that low levels of 25(OH)D3, 24,25(OH)_2_D3 and VDMR have significant potential for prediction of CRC development, while 25(OH)D3 retained its significance after the adjustment for traditional risk factors (age, male sex and BMI). However, after the inclusion of TC concentration in the Model 2, 25(OH)D3 lost its predictive potential. On the other hand, TC concentration was recognized as potential significant predictor of CRC in combination with traditional risk factors and 25(OH)D3 (OR: 0.417; CI: 0.267-0.650; P<0.001), 24,25(OH)_2_D3 (OR: 0.409; CI: 0.262-0.637; P<0.001), and VDMR (OR: 0.404; CI: 0.259-0.629; P<0.001).

**Table 5:** Logistic regression analysis for evaluation of parameters of vitamin D status in prediction of risk for CRC development Model 1: adjustment was made for age, gender (m/f) and BMI. Model 2: adjustment was made for age, gender (m/f), BMI and TC.

Univariate logistic regression			
Parameter	OR	95% CI (OR)	P
7-DHC (μmol/L)	0.926	(0.762–1.124)	0.437
25(OH)D3 (ng/mL)	0.934	(0.892–0.979)	0.004
24,25(OH)_2_D3	0.701	(0.514–0.956)	0.025
VDMR	0.769	(0.604–0.980)	0.034
VDBP (_m_g/mL)	0.997	(0.993–1.001)	0.115
Multivariate logistic regression			
Model 1			
Parameter	OR	95% CI (OR)	P
25(OH)D3 (ng/mL)	0.943	(0.893–0.996)	0.035
24,25(OH)_2_D3	0.714	(0.498–1.025)	0.068
VDMR	0.837	(0.613–1.144)	0.264
Model 2			
Parameter	OR	95% CI (OR)	P
25(OH)D3 (ng/mL)	0.967	(0.912–1.025)	0.263
24,25(OH)_2_D3	0.807	(0.558–1.168)	0.256
VDMR	0.936	(0.669–1.310)	0.701

Similar analysis was performed to explore potential of vitamin D status parameters in prediction of tumor grade. Patients are stratified in two cohorts: subjects with low grade tumor (grade I) and subjects with high grade tumor (grade II + grade III). Our results (Table 6) demonstrated that only 25(OH)D3 was a significant risk factor for high grade carcinoma development. In contrast to previous analysis, the observed significance retained after adjustment for other cofounders (age, male sex and BMI) and even after inclusion of TC in the model.

**Table 6:** Parameters of vitamin D status in prediction of high grade tumor development Model 1: adjustment was made for age, gender (m/f) and BMI. Model 2: adjustment was made for age, gender (m/f), BMI and TC.

Univariate logistic regression			
Parameter	OR	95% CI (OR)	P
7-DHC (μmol/L)	1.040	(0.811–1.334)	0.756
25(OH)D3 (ng/mL)	0.911	(0.845–0.983)	<0.05
24,25(OH)_2_D3	0.548	(0.301–0.999)	0.050
VDMR	0.734	(0.527–1.023)	0.068
VDBP (μg/mL)	0.997	(0.992–1.002)	0.281
Multivariate logistic regression			
Model 1			
Parameter	OR	95% CI (OR)	P
25(OH)D3 (ng/mL)	0.895	(0.822–0.975)	<0.050
Model 2			
Parameter	OR	95% CI (OR)	P
25(OH)D3 (ng/mL)	0.898	(0.823–0.979)	<0.050

## Discussion

In this study, we demonstrated that concentrations of 7-DHC, 25(OH)D3 and VDMR were de-creased in CRC patients. 25(OH)D3 emerged as the most prominent marker of changed vitamin D status in CRC patients, as well as the molecule with the highest potential for prediction of CRC development. However, its predictive potential was abolished when TC, as a routine lipid status marker, was included in the analysis.

It is well known that status of vitamin D is diminished in patients with CRC [3]. Our results (Table 1) support these findings. We observed a decrease in concentrations of 7-DHC, 25(OH)D3 and VDBP, but no differences between patients and controls were recorded for 24,25(OH)_2_D3 (Table 1). It is widely accepted that seasonal variations in sun exposure are major contributors to the vitamin D status [13], which was also confirmed by the results in our healthy cohort (Table 2). However, when we analyzed vitamin D metabolites in CRC patients, we did not find any differences that could be attributed to the seasonal variations in sun exposure. Therefore, our results suggest that lower vitamin D status in CRC patients is more likely a consequence of processes related to the disease itself, than a result of reduced sun exposure.

Metabolic processing of vitamin D is complex and not solely limited to liver and kidney. Instead, extrarenal tissues, including colon, are active sites of vitamin D metabolism [14]. Namely, it has been demonstrated that both CYP27B1, an enzyme responsible for synthesis of active vitamin D form, and CYP24A1, responsible for synthesis of inactive 24,25(OH)2D3 metabolite, are present in colonocytes and regulated by the level of cell's differentiation [14]. Since previous researches confirmed enhanced activity of CYP24A1 in less differentiated colon cancer cells [14] [15], it would be expectable to find increased concentrations of 24,25(OH)2D3 in systemic circulation of CRC patients. However, our results did not show any differences in 24,25(OH)2D3 serum levels between patients and controls (Table 1). Yet, it is important to notice that evidences regarding increased CYP24A1 and 24,25(OH)_2_D3 are derived from studies on cancer tissue and cells, but not from analyses in serum. Since blood levels of vitamin D metabolites are a reflection of metabolic processes in numerous tissues, but principally in liver and kidney, the influence of altered metabolism in cancer cells might not be sufficient to change serum concentration of a particular metabolite. However, eventual broad use of vitamin D meta-bolites for prediction and prognosis of CRC development would likely be oriented toward serum or plasma samples. Our results (Table 1) suggest that 24,25(OH)_2_D3 might not be a reliable marker for these purposes. In addition, although 24,25(OH)_2_D3, alongside with 7-DHC were recognized as independent predictors of CRC in an univariate analysis, adjustment for age, gender and BMI abolished their prognostic capacity (Table 5), thereby confirming the above mentioned presumption. On the other hand, VDMR was significantly lower in patients (Table 1), but such finding most likely reflected the presence of significant differences in 25(OH)D3 concentrations, thus emphasizing the importance of this vitamin D metabolite.

It has been shown that vitamin D metabolism in malignant cells is influenced by the extracellular availability of its active form and subsequent interaction with vitamin D receptors [16]. Also, previous studies have demonstrated that the activity of CYP27B1 and CYP24A1 depends on availability of substrate 25(OH)D3 [17] [18]. Accordingly, overall amount of 25(OH)D3 is responsible for local activation and effects of vitamin D. In our study, serum 25(OH)D3 and 7-DHC were significantly lower in CRC patients (Table 1), thereby setting prerequisites for later defective vitamin D activation and biological function in extrarenal tissues. In parallel with low levels of vitamin D precursors, we also observed a decrease in TC, LDL-C and HDL-C levels (Table 1). These findings could be of particular importance, since vitamin D and cholesterol share a common precursor: 7-DHC. Previous studies of serum lipid levels in CRC are inconsistent, but the findings similar to ours were reported in the study of Abaza et al. [18]. Apart from poor nutritional status frequently seen in CRC [19], which could be one of the reasons for decreased cholesterol synthesis in the liver, it has been recently proposed that reprogramming of lipid metabolism could be responsible for the observed decrease in serum lipid status markers in various types of cancer [19] [20]. Namely, it has been demonstrated that cancer cells extensively accumulate and use cholesterol [21] [22], while such increased needs are satisfied by either upregulated endogenous synthesis [23], or enhanced uptake of circulating cholesterol [24] [25]. Such rearrangement of cholesterol synthesis and cellular accumulation could have important consequences regarding vitamin D status. It has been proposed that 7-DHC is placed in the center of metabolic shift between cholesterol and vitamin D synthesis pathways [7]. In our study, lower levels of 7-DHC were recorded in CRC patients (Table 1). Thus, it is possible that in conditions of increased utilization of cholesterol by malignant cells and consequent generalized direction of biosynthesis towards cholesterol, vitamin D synthesis is compromised. In confirming such assumption, we observed strong positive correlation of serum levels of TC, LDL-C and HDL-C with 7-DHC and analyzed vitamin D3 metabolites, but only in a group of CRC patients. These findings suggest that decreased serum cholesterol levels, which are indicative for increased cholesterol utilization by malignant cells, are associated with diminished vitamin D synthe-sis and further metabolic transformation. Previously, Bogh et al. [25] have demonstrated that elevation of 25(OH)D3 level after sun exposure was in positive correlation with TC concentration. Thus, local production of vitamin D in the skin can also be compromised in the conditions of decreased serum TC in CRC. Due to generalized lack of circulating cholesterol, as seen in CRC, and consequent disabled uptake by the other body structures apart from malignant tissue, it would be reasonable to expect that biosynthesis pathways in keratinocytes would likely be directed towards cholesterol, rather than towards vitamin D. Taken all together, our results might indicate that decreased levels of vitamin D, regularly seen in CRC, are in the first place a consequence of disturbed lipid profile. Indeed, when we divided CRC patients according to TC concentrations (Table 4), we found lower values of 7-DHC, 25(OH)D3 and even 24,25(OH)_2_D3 in subjects with low TC, thereby confirming the impact of circulating cholesterol on all aspects of vitamin D metabolism.

Finally, when we explored independent potential of vitamin D metabolites in predicting the risk for CRC development (Table 5), serum levels of 25(OH)D3 emerged as the most potent marker, whose relevance was retained even after the adjustment for well-known contributors of CRC development. However, inclusion of TC concentration to the designed model, eliminated the independent impact of decreased 25(OH)D3 on CRC development, which is in line with the postulated role of TC in directing of vitamin D metabolism. Interestingly, low serum TC was recognized as an independent predictor of CRC, emphasizing the role of lipid alterations in etiopathogenesis of this disease. The role of lipids in cancer development was neglected for a long time, but recent research shed light on this topic [19]. We have previously demonstrated changed activity of lipid transfer proteins [11] and alterations of lipoprotein subclasses [12] in CRC patients. Possible influence on vitamin D metabolites demonstrated herein can present an additional effect of disturbed lipid homeostasis during the development and progression of CRC.

Nevertheless, the role of vitamin D itself on the tumor progression cannot be neglected. Namely, we found a decrease in serum 25(OH)D3 levels across tumor grades in parallel with an increase in 24,25(OH)_2_D3 in grade II CRC (Table 3). The lack of significant differences during comparison of grade III subgroup with other subjects is most likely a consequence of smaller number of participants in this group. It is well known that vitamin D enhances cell differentiation [1] [2], so the observed decrease of 25(OH)D3 in patients with poorly differentiated cancer is expected. More importantly, low 25(OH)D3 level was revealed as independent predictor of high grade CRC, even after adjustment for other risk factors, including TC concentration (Table 6). Therefore, our results suggest that 25(OH)D3 could be useful as potential serum marker of increased risk for high grade CRC, which should be further explored in future studies. Data about the association between vitamin D and tumor grade in CRC are scarce. Yet, it has been recently demonstrated that expression of nuclear vitamin D receptors gradually decreases during the progression of low grade adenoma towards high grade adenoma and CRC [26]. Our findings regarding serum vitamin D levels are in agreement with these results. It also should be noticed that determination of serum 25(OH)D3 is a simple and inexpensive procedure, implying that vitamin D might be considered as easily available and potentially useful indicator of tumor grade in CRC patients.

It should also be mentioned that we observed significantly lower VDBP levels in CRC patients when compared to healthy participants (Table 1). Such results were expectable since VDBP is primarily synthesized by the liver and it is well documented that tumor-associated inflammation and production of cytokines can decrease hepatic protein synthesis, while on the other hand, malignant cells can increasingly uptake serum proteins [27]. Altogether, these processes result in decreased total serum protein which was also seen in our study (Table 1). However, our results did not demonstrate significant independent contribution of VDBP to the modulation of risk for CRC development (Table 5), which is in line with previous researches [27] [28], thus suggesting that the impact of VDBP during CRC progression is more likely indirect and related to vitamin D.

Several limitations should be emphasized. Due to increased prevalence of CRC in older subjects and limited possibilities to involve a sufficient number of elderly healthy volunteers, patients and controls were not completely matched by age. Although older age is recognized as a significant contributor to lower vitamin D status, in this study we found no differences in vitamin D metabolites among different age groups. However, our results should be further evaluated in groups of patients and controls matched by age.

Patients and controls could not be matched entirely by age for the purpose of this study. Additionally, since this is an observational study, we could not fully explore the presumed causality between decreased TC level and minimized vitamin D synthesis. Future researches for verifying our hypothesis and elucidating possible mechanisms of these interactions are needed. Next, cross-sectional nature of our study prevented us from drawing conclusions regarding the role of observed associations during the progression of CRC. Forthcoming prospective studies should address this topic.

## Conclusions

In conclusion, our results demonstrated decreased levels of 7-DHC, 25(OH)D3 and VDMR in patients with CRC. In addition, we observed a significant positive association between vitamin D status and serum lipid markers. Although 25(OH)D3 was revealed as significant marker of CRC development, the obtained results suggested profound impact of serum cholesterol on the levels of vitamin D metabolites and give a new perspective for understanding the vitamin D deficiency in CRC, and possible improvement of patient management strategies.

Funding: This study was financially supported by a grant from the Ministry of Education, Science and Technological Development, Republic of Serbia (Project No. 175035).

## Conflict of interest statement

The authors state that they have no conflicts of interest regarding the publication of this article.

## List of abbreviations

CRC, colorectal cancer; 7-DHC, 7-dehydrocholesterol; VDMR, vitamin D metabolic ratio; DHCR7, 7- DHC-reductase; BMI, body mass index; TC, total cholesterol; LDL-C, low-density lipoprotein cholesterol; HDL-C, high-density lipoprotein cholesterol; TG, triglycerides; VDBP vitamin D binding protein; MTBE, methyl-tert-buthyl ether; MRM, multiple-reaction-monitoring; MMI/APCI, multi-mode ionization/ atmospheric pressure chemical ionization; OR, odds ratio; CI, confidence interval.
